# Retrospective Investigation of an Influenza A/H1N1pdm Outbreak in an Italian Military Ship Cruising in the Mediterranean Sea, May-September 2009

**DOI:** 10.1371/journal.pone.0015933

**Published:** 2011-01-20

**Authors:** Mario Tarabbo, Daniele Lapa, Concetta Castilletti, Pietro Tommaselli, Riccardo Guarducci, Giuditta Lucà, Alessandro Emanuele, Onofrio Zaccaria, Vincenzo F. P. La Gioia, Enrico Girardi, Maria R. Capobianchi, Giuseppe Ippolito

**Affiliations:** 1 Inspectorate of Health, ITA Navy, Rome, Italy; 2 Laboratory of Virology, National Institute for infectious Diseases “L. Spallanzani”, Rome, Italy; 3 ITA Navy, Rome; 4 Military Hospital, ITA Navy, Taranto, Italy; 5 Frigate “Scirocco”, ITA Navy, Italy; 6 Medical Situation Awareness Office, ITA Defence General Staff, Rome, Italy; 7 Department of Epidemiology, National Institute for Infectious Diseases “L. Spallanzani”, Rome, Italy; 8 Scientific Direction, National Institute for Infectious Diseases “L. Spallanzani”, Rome, Italy; University of Liverpool, United Kingdom

## Abstract

**Background:**

Clinical surveillance may have underestimated the real extent of the spread of the new strain of influenza A/H1N1, which surfaced in April 2009 originating the first influenza pandemic of the 21^st^ century. Here we report a serological investigation on an influenza A/H1N1pdm outbreak in an Italian military ship while cruising in the Mediterranean Sea (May 24-September 6, 2009).

**Methods:**

The contemporary presence of HAI and CF antibodies was used to retrospectively estimate the extent of influenza A/H1N1pdm spread across the crew members (median age: 29 years).

**Findings:**

During the cruise, 2 crew members fulfilled the surveillance case definition for influenza, but only one was laboratory confirmed by influenza A/H1N1pdm-specific RT-PCR; 52 reported acute respiratory illness (ARI) episodes, and 183 reported no ARI episodes. Overall, among the 211 crew member for whom a valid serological result was available, 39.3% tested seropositive for influenza A/H1N1pdm. The proportion of seropositives was significantly associated with more crowded living quarters and tended to be higher in those aged <40 and in those reporting ARI or suspected/confirmed influenza A/H1N1pdm compared to the asymptomatic individuals. No association was found with previous seasonal influenza vaccination.

**Conclusions:**

These findings underline the risk for rapid spread of novel strains of influenza A in confined environment, such as military ships, where crowding, rigorous working environment, physiologic stress occur. The high proportion of asymptomatic infections in this ship-borne outbreak supports the concept that serological surveillance in such semi-closed communities is essential to appreciate the real extent of influenza A/H1N1pdm spread and can constitute, since the early stage of a pandemic, an useful model to predict the public health impact of pandemic influenza and to establish proportionate and effective countermeasures.

## Introduction

During April-May 2009, a new strain of influenza A rapidly spreading from Mexico all around the world, originated the first influenza pandemic of the 21^st^ century. Early data from Mexico suggested that this new pandemic virus strain (A/H1N1pdm) had a high infection rate in younger age groups, and high case-fatality ratio, at least in some particular risk groups [1]–[3].

However, most of these inferences derived from confirmed cases that rely on laboratory results showing the presence of influenza virus genome in the respiratory tract of the affected individuals, selected on the basis of a strict case definition that tended to exclude less severe cases.

Subsequent reports suggested that the actual burden of the infection had been largely underestimated, while the clinical severity has been overestimated, and that serological investigation may be helpful to establish a more accurate estimate of the infection rate, especially since a substantial proportion of influenza infections are asymptomatic [4]. In fact, mild afebrile illness has been described in 8 to 32% of infected persons [5]. Consistently, seroincidence has been reported to be 10 times higher than estimates from clinical surveillance [6]–[7], and a high proportion (36%) of A/H1N1pdm seroconverters are asymptomatic, similarly to seasonal influenza [8].

In keeping with these observations, estimates of the secondary attack rate have shown wide variability, depending on the methods used. For instance, estimates of secondary attack rate among household contacts reported in different studies ranged from 4% to 36%, with lower figures when estimated through PCR confirmation of clinically apparent disease and higher figures when estimated through retrospective serology [1], [ 8]–[9].

Some studies have been conducted on the crew members of military ships that represent a particular semi-closed community of individuals with rather homogeneous demographic characteristics. From these reports, 7.3%–12% shipmates have contracted the pandemic influenza on the basis of symptoms (ILI) [10]–[12]. In the studies from Almond and from Crum-Cianflone the rate of infection was lower (respectively 3.13% and 8%) if estimated through PCR [10]–[11]. A substantially higher proportion of infections, was reported for another shipborne outbreak, where 22% of shipmates acquired the infection (symptomatic, PCR-positive) in a Peruvian Navy ship docked at San Francisco during June 2009 [13]. From these studies, it was not possible to estimate the real extent of the outbreaks, as the starting criterion for case definition was the presence of ILI, therefore both afebrile respiratory illnesses and asymptomatic infections have been disregarded. In fact, based on seroconversion rate, a study conducted in military personnel from Singapore estimated an infection rate of 29.4% [14].

Here we report a retrospective serological investigation on the crew members of a military ship that left Italy on May 24, 2009 and stopped at several Mediterranean ports before ending the cruise. During the cruise, several cases of acute respiratory illnesses (ARI) occurred, but only 2 met the ILI case definition. Laboratory confirmation of influenza A/H1N1pdm infection was carried out at the closest harbour hospital only in these 2 patients, resulting in one confirmed case. Both cases were kept in isolation in this hospital, until the resolution of symptoms. Considering the reported range of the attack rate for influenza [10]–[15], the number of cases fulfilling the case definition of ILI seemed to be rather low, and, in addition, it was surprising that in a quasi-closed community only one case of confirmed A/H1N1pdm infection occurred during the cruise timeline. Therefore, once the ship was on the way back to Italy, it was decided to carry out a serological survey to try to estimate the real extent of the outbreak among crew members.

## Results

### Description of the shipborn outbreak

Scirocco, an Italian military ship, sailed from Taranto (Italy) on May 24, 2009. The ship had a crew of 237 members (93.2% men) with a median age of 29 years (interquartile range 26–35 years). One hundred and twenty nine (54.4%) crewmembers had been vaccinated against seasonal influenza during the 2008–2009 season. Soon after the departure, following the alarm launched by Italy to the Ministry of Health, surveillance of A/H1N1pdm influenza was instituted on the ship and crewmen were encouraged to report to infirmary any acute onset of respiratory symptoms. For the purpose of surveillance, a case of suspected A/H1N1pdm was defined as fever ≥38°C plus one or more respiratory symptoms (cough, sore throat, rhinorrhea) and one or more general symptoms (limb/joint pain, headache, malaise), according to the case definition in use in Italy at that time (http://www.normativasanitaria.it/normasan-pdf/0000/29528_1.pdf). Respiratory illnesses, which did not fulfill the case definition, were classified as acute respiratory illness (ARI).

The ship reached Beirut, Lebanon, on May 27, 2009 and stopped there for 3 days. The ship continued her cruise in the Mediterranean Sea with six three-day stops in Beirut between June 22 and August 31. The ship also stopped in Lymassol, Cyprus on June 8 to 10, 2009, and in Mersin Turkey, July 1 to 6, 2009. When the ship was at moorings, crewmen were allowed to go ashore for protocol or visiting activities, on a rotational basis (one-third remaining on board).

At the time the ship left Italy, there were 19 laboratory-confirmed A/H1N1pdm influenza cases reported in Italy (data available from Italian Ministry of Health http://www.sanita.it/Malinf_gestione/Rischi/documenti/137-09.pdf). A/H1N1pdm influenza cases were also reported in Lebanon (n = 3) (data available from WHO http://www.who.int/csr/don/2009_06_03/en/index.html), Cyprus (n = 1) (data available from WHO http://www.who.int/csr/don/2009_06_10a/en/index.html) and Turkey (n = 40) (data available from WHO http://www.who.int/csr/don/2009_07_03/en/index.html) at the time the ship docked there for the first time.

On August 9, 2009 (week 32) a crewmember presented to infirmary reporting fever (>38°C), malaise and rhinorrhea. A rapid influenza A+B test was performed (Quickvue, Quidel, CA, USA) which resulted positive, and the seaman was started on Oseltamivir (TAMIFLU 75 mg, 2 tablets/die). The next day the patient was admitted to the infectious disease unit at Rafik Hariri University Hospital in Beirut, were a pharyngeal swab resulted positive to influenza A A/H1N1pdm by the RT-PCR [16]. The patient was kept in isolation at Hariri Hospital until August 17 when he was discharged and returned to the ship.

Another crewmember, who had presented with rhinorrea and malaise on August 8 (week 32), 2009, developed fever (>38°C) on August 11, fulfilling the suspected case definition. Thus a rapid influenza A+B test was performed which resulted positive. He was started on Oseltamivir and was admitted to Hariri hospital. A pharyngeal swab was negative for influenza A A/H1N1pdm by RT-PCR. The patient was discharged from the hospital on August 17 and returned to the ship.

**Figure 1:**
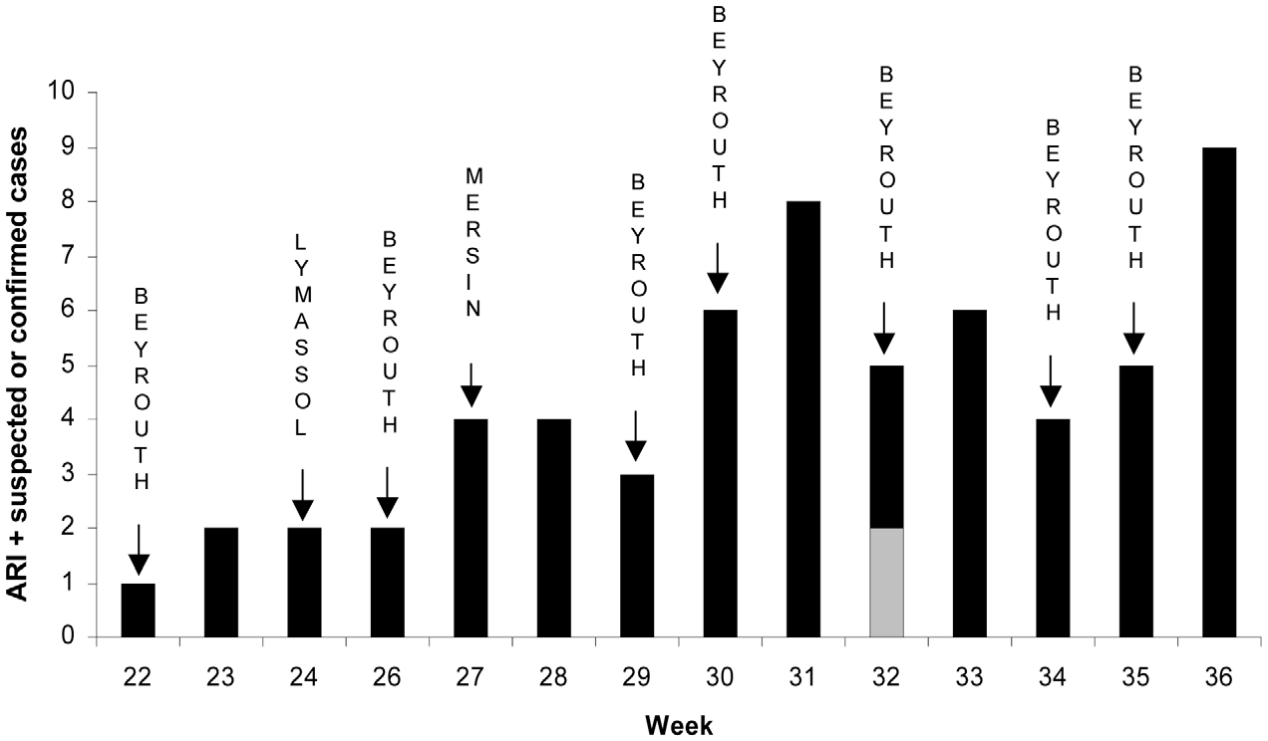
Distribution of cases according to the time of symptoms onset. Black bar: acute respiratory illness (ARI) episodes; gray bars: episodes fulfilling suspected A/H1N1pdm case definition. The arrows indicate the arrival to the stopover port. Location of docking sites: Beirut, Lebanon; Lymassol, Cyprus; Mersin, Turkey.

No other cases fulfilling the case definition for suspected A/H1N1pdm influenza were recorded during the cruise duration (from May 24, week 21, to September 6, week 36). However 59 additional episodes of ARI occurred in 52 crew members after the ship departure. None of these cases was tested for the presence of influenza A/H1N1pdm virus in respiratory secretions. The distribution of ARI and suspected or confirmed cases according to the time of symptoms onset is shown in **Figure 1**. During the first 4 weeks of the cruise 1 or 2 ARI cases per week were recorded. The number of cases increased thereafter, to reach 8 and 9 cases per week at weeks 31 and 36, respectively.

### Establishment of serological cut offs and estimate of seroprevalence in the civilian population matching the personal data of military shipmates

Since pre-outbreak serum samples of ship military personnel were not available, our estimate of the infection rate must be solely based on the presence of antibodies at the end of the cruise. To establish an appropriate criterion to retrospectively diagnose A/H1N1pdm infection, we used paired acute and convalescent serum samples from patients with A/H1N1pdm infection confirmed by RT-PCR (group 1) taken ≤4 days post-symptom onset (T0) and 2 or 3 weeks later (T1). In addition, to obtain data on the pre-pandemic prevalence of antibodies to A/H1N1pdm, we used residual serum samples, stored in the local biorepository, that had been submitted to our laboratory for HIV antibody screening and resulted HIV-negative in September 2008, from males matching the age group represented in the study population (20–45 years old, n = 50, group 2). The serum samples were rendered anonymous before testing for influenza antibodies. To establish the contemporary prevalence of antibodies to A/H1N1pdm, another group of serum samples from a similar population, collected on September 2009 (n = 50, group 3), was selected. In addition, a third equivalent group of serum samples collected two months after the pandemic influenza peak in Italy (February 2010, n = 50, group 4), was selected.

All these samples were tested with HAI and CF assay, and an antibody titer ≥1∶10 in both HAI and CF assays was established as a cut-off for seropositivity to influenza A/H1N1pdm. The results obtained on these four sets of samples, shown in **Table 1**, support the adequacy of the adopted criterion to retrospectively identify the A/H1N1pdm infections.

**Table 1:** Proportion of serum samples with contemporary HAI and CF titers ≥1∶10 in 4 control groups.

	Time of collection	Frequency of individuals showing both HAI and CF titers ≥1∶10
**Group 1** **(A/H1N1pdm-infected patients)**	**T0**	0%
	**T1**	85.7%
**Group 2**	**September 2008**	10.2%
**Group 3**	**September 2009**	8.2%
**Group 4**	**February 2010**	37.7%

**Group 1: T0 and T1:** paired acute and convalescent serum samples from patients with A/H1N1pdm infection confirmed by RT-PCR.

**Group 2:** serum samples submitted to our laboratory for HIV antibody screening and resulted HIV-negative in September 2008, from males matching the age group represented in the study population.

**Group 3:** serum samples submitted to our laboratory for HIV antibody screening and resulted HIV-negative in September 2009, from males matching the age group represented in the study population.

**Group 4:** serum samples submitted to our laboratory for HIV antibody screening and resulted HIV-negative, 2 months after the pandemic influenza peak in Italy (February 2010).

### Serological survey on the crew members

Serum samples from the study subjects were collected aboard on September 6, 2010, just before the end of the cruise. A serum sample was available for 216 of the 237 crew members; for 5 samples the results of serology were not interpretable because of unspecific reaction in HAI test. Overall a positive serology was found for 83 of the 211 crew member for whom a valid result was available (39.3%). As shown in **Table 2**, the proportion of individuals testing positive was higher among individual aged less than 40 compared to older crewmen, although this difference was not significant, while no difference was found according to rank. No significant difference was found when comparing prevalence recorded in specific living quarters or working areas, unless considering their crowding. In fact, those persons who were in living quarters with 1–2 persons were significantly less likely to test positive when compared to the personnel of more crowded living quarters, and a similar trend was found when considering the number of persons in working areas (**Table 2**).

**Table 2:** Results of influenza A/H1N1pdm serology in 211 crew members of the Italian military ship Scirocco.

Characteristics	Positive/total (%)	Odds Ratio(95% confidence interval)	p
**Age**			
≥40 years	10/34 (29.4)	ref	
30–39 years	30/71 (42.3)	1.76 (0.73 – 4.21)	0.27
20–29 years	43/106 (40.6)	1.64 (0.71–3.77)	0.24
**Rank**			
Commissioned officers	14/29 (48.3)	ref	
Warrant officers	32/84 (38.1)	1.54 (0.67 – 3.55)	0.31
Petty officers/enlisted personnel	37/98 (37.8)	1.01 (0.56 – 1.85)	0.96
**Number of persons in living quarter**			
1–2	5/27 (18.5)	ref	
3–9	16/35 (45.7)	3.70 (1.14–12.02)	0.03
≥10	62/149 (41.6)	3.17 (1.13– 8.73)	0.03
**Number of persons in working area**			
1–5	7/28 (25.0)	ref	
6–10	20/48 (41.7)	2.14 (0.76 – 6.0)	0.14
>10	56/135 (41.5)	2.13 (0.85 – 5.34)	0.11
**Previous seasonal influenza vaccination**			
Yes	37/96 (38.5)	ref	
No	46/115 (40.0)	1.06 (0.61–1.85)	0.83

The proportion of positive results was similar in individuals who received or did not receive seasonal influenza vaccination in the previous year.

All the individuals who reported an ARI episode or suspected or confirmed A/H1N1pdm influenza had available serology results. Both individuals who fulfilled the surveillance case definition for influenza tested positive, as did 4/7 (57.10%) individuals with two episodes of ARI and 21/45 (46.7%) of individuals who reported a single ARI episode (**Table 3**). Overall, the proportion of individuals testing positive was higher among those who reported ARI or suspected/confirmed A/H1N1pdm influenza (27/54, 50.0%) compared to those who did not (58/157, 36.9%) although the difference was not statistically significant (p = 0.12).

**Table 3:** Results of influenza A/H1N1pdm serology in 211 crew members of the Italian military ship Scirocco, according to respiratory illness reported during the cruise.

Type of illness	Positive/total (%)
Confirmed/suspected influenza A/H1N1pdm	2/2 (100)
Acute respiratory illness (1 Episode)	21/45 (46.7)
Acute respiratory illness (>1 Episode)	4/7 (57.1)
No acute respiratory illness	58/157 (36.9)

**Figure 2:**
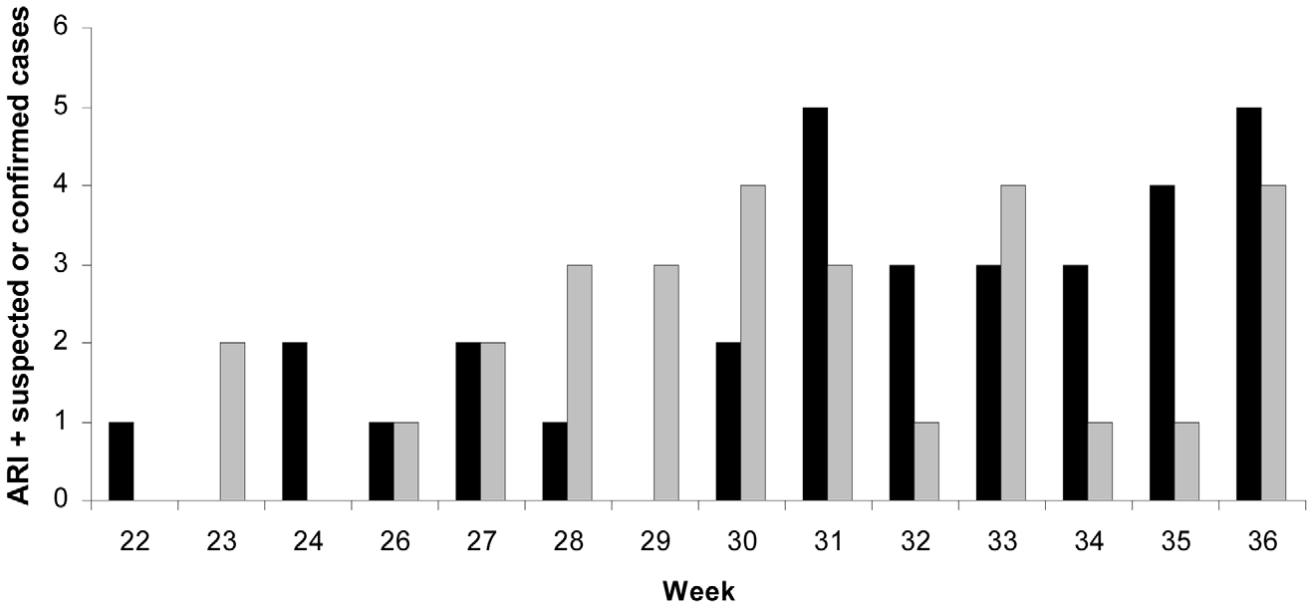
Distribution of acute respiratory illness (ARI) and suspected A/H1N1pdm cases, according to the time of symptoms onset and to the results of Influenza A/H1N1pdm-specific serological tests. Black bars: seropositives; gray bars: seronegatives.

**Figure 2** shows the distribution over time of ARI and suspected or confirmed cases according to the results of serological tests. Cases occurring in individuals who tested positive increased between weeks 31 and 36. However, due to the short time lapse between the last recorded episodes and the serum sampling time, it is reasonable to expect that a significant proportion of individuals who could have been infected after week 35 would result serologically negative.

It is noteworthy that, among the patients who tested serologically positive to A/H1N1pdm, the first ARI appeared on May 25, i.e. two days after the cruise start and two days before the first stop of the ship (Beirut), in a patient who did not show any subsequent ARI episode, suggesting that this first (possibly index) case could have acquired the infection in Italy.

## Discussion

It is well known that the serological response to the infection with a given influenza subtype is influenced by the extent of cross-reaction with heterologous subtypes, previous exposure to related strains, individual variability and age. In addition, the measure of antibody response is prone to variability due to the experimental procedures, such as type of red blood cells or virus preparation. Several studies have considered a HAI titer in the range of ∼1∶40 for their conclusions, as the scope was to establish the level of pre-existing protective immunity, while only few studies reports lower thresholds (e.g.1∶10), as indicators of previous exposure to related strains of influenza virus. Considering the protective threshold, in Italy the pre-pandemic frequency of antibodies against A/H1N1pdm in the general population is in the range of 6–7% individuals aged 0–55 years, and increases to 22% in over 65 [17]. In UK, a protective titer has been detected in 9.8% of individuals aged 25–49 [7], while 18.1% shows antibodies titered 1∶8 in the pre-pandemic era. In another study, conducted in Finland, 0.8% of individuals aged 20–39 show protective antibodies, and 2.5% showed titers of 1∶10 [18]. The pre-pandemic prevalence of HAI antibodies in military personnel was 15% with at 1∶10 and 9.4% at 1∶40 [14]. In residual hospital serum samples from persons aged 18–24 years the pre-pandemic seroprevalence (1∶40) was 6% [19].

The aim of the present study was to establish retrospectively the rate of infection in a close community for which pre-infection serum samples were not available. The combined serological approach, based on the contemporary presence of HAI and of CF antibodies at a titer ≥1∶10, was established on the basis of a preliminary analysis conducted in patients who seroconverted by definition (infected patient's population, group 1), and was validated in three sentinel groups, representing, respectively, age matched male individuals sampled before the pandemic (group 2), at the time of the cruise end (group 3) and after the 2009 pandemic influenza wave (group 4). In fact, the low HAI titer threshold allowed us to maximize the sensitivity, and the contemporary presence of CF was used to increase the specificity of our infection criterion, as this type of antibodies is commonly recognized as indicator of recent infection [20]–[21].

On the whole, the data obtained on a representative adult population matching the demographical characteristics of our study population indicates that, indeed, the rate of serological positivity against A/H1N1pdm in the pre-pandemic era is about 10%, confirming previous literature data.

In the time frame overlapping with that considered in the study, this proportion do not significantly increase in the control population.

Thus, assuming a similar background of 10% of seropositivity in the study population, we may infer that about 30% of the sailors actually had acquired the infection during the cruise. The association of the positivity rate with crowding of living quarters supports this hypothesis.

This estimate is in keeping with that from a study conducted in military personnel from Singapore (29.4%), based on seroconversion rate over a 3 months period during 2009 [14].

In the Scirocco ship, only two of the seropositives showed clinical signs consistent with the case definition, while about half showed mild symptoms and the remaining half remained fully asymptomatic during the cruise.

These results are in apparent contradiction with those from other shipborne outbreaks, where higher proportion of febrile infections has been reported [10]–[13]. The reasons for such differences are unclear, although some discrepancies in clinical surveillance systems may be at least in part accounting for. Nevertheless, our results suggest that the introduction influenza A/H1N1pdm in a close community may result in a small number of clinically relevant diseases in spite of a wide spread, detected by serological investigation.

Our data also suggest that previous vaccination against seasonal viruses was not protective against A/H1N1pdm infection, in agreement with previous reports performed in similar settings [11], [13].

The present study presents some limitation. First, pre-pandemic serum samples of the crewmen were not available; therefore our estimate of the baseline seroprevalence is only speculative, leading to a possibly imprecise estimate of proportion of peoples who contracted the infection during the cruise. Second, according to the surveillance protocol, afebrile or low grade fever cases were not tested by PCR, so it is not possible to demonstrate that cases occurring in patients who tested seropositive at the end of cruise were actually attributable to influenza A/H\N1pdm infection.

The results of the present study may be relevant for planning public health strategies in the context of early pandemic. In fact, clinical surveillance criteria established during the early phases of an evolving pandemic may prove inadequate to monitor the actual spread and the severity of the phenomenon, and need timely update to provide a realistic estimate. In this context, serosurveillance data, particularly from semi-closed communities, may be crucial in order to timely define the real spectrum of clinical presentation and the possible public health impact, essential to identify and implement adequate control measures. As final consideration, biorepositories may represent a valuable resource to help define the pre-pandemic population immunity and to monitor the changes in the sero-prevalence, providing unbiased collection of samples supplied with demographic, epidemiological and clinical information.

## Materials and Methods

### Source of samples

#### Crew members

The study was performed among 216 crew members of the Italian military ship Scirocco. A sample of about 5 ml of venous blood was taken at the end of cruise, immediately before the personnel left the ship. The study has been approved by the Ethics Committee of the National Institute for Infectious Diseases “L. Spallanzani”. All study participants provided written informed consent.

### Virus stock preparation

A/H1N1pdm influenza virus (kindly provided by Prof. A. Azzi, Florence, Italy) was amplified on Madin-Darby Canine Kidney cells genetically modified to over-express α-2,6-linked sialic acid (MDCK SIAT1, kindly provided by F. Baldanti, Pavia, Italy) in Dulbecco's-modified-Eagle's medium (D-MEM) containing 2 µg/ml of TPCK-treated trypsin (SIGMA) at 35°C in a 5% CO_2_ humidified atmosphere. The MDCK SIAT1 cell line has been previously described [22]. The haemagglutination titer of the virus stock was determined using group 0 fresh human blood red cells according to the WHO protocol [23].

The virus was inactivated by exposing to UV lights for 10′ and stored at –80°C until use. Complete inactivation of UV-exposed virus was checked by infecting MDCK SIAT1 monolayers with undiluted preparation and by back titrating the infectivity after 5 days of incubation.

### Serologic tests

To retrospectively identify the A/H1N1pdm infections, the antibody titer established by Haemagglutination Inhibition (HAI) and Complement Fixation (CF) assays was determined. The choice of performing in parallel FC and HAI assay was motivated by the transient expression of the fixing complement antibodies, which renders the test more useful in studies of recent infections [20]–[21], and may partially correct the seroprevalence values estimated on the basis of HAI, that shows a variable extent of background positivity [7]. As challenge in both assays an UV-inactivated influenza A/H1N1pdm virus preparation was used. HAI assays were performed in V-bottom 96-well plates using group 0 fresh human blood red cells [23]. All specimens were tested in serial twofold dilutions (from 1∶10 up to 1∶320). CF test was done by a standard Kolmer microthecnique [20] using the same inactivated virus preparation. Standard reagents for the development of the CF reaction were from commercial source (Institut Virion/Serion GmbH, Germany). Serial serum dilutions (1∶10 up to 1∶80) were tested. Negative and high-positive controls were included in each run of HAI and CF tests.

For computational purpose, a value of 1∶5 was assigned to the samples resulting CF- or HAI-negative at 1∶10.

The reciprocal of the dilutions of the HAI and CF were transformed to log_2_ for statistical evaluation.

### Biosafety Laboratory Facilities

All experiments with live A/H1N1pdm were conducted by using Biosafety Level 3-plus (BSL3+) containment procedures [24]. All the investigators were required to wear appropriate masks with HEPA filters.

### Statistical analysis

Standard univariate methods were used to assess the association between individuals' characteristics and serology results.
